# Supplementary material for: Diversity-decomposition relationships in forests worldwide
Source: eLife. 2020 Jun 26;9:e55813. doi: 10.7554/eLife.55813 (PMC7402676; doi:10.7554/eLife.55813)
Supplement: Transparent reporting form [file elife-55813-transrepform.docx]

***eLife’s* transparent reporting form**

We encourage authors to provide detailed information *within their submission* to facilitate the interpretation and replication of experiments. Authors can upload supporting documentation to indicate the use of appropriate reporting guidelines for health-related research (see [EQUATOR Network](http://www.equator-network.org/%20)), life science research (see the [BioSharing Information Resource](https://biosharing.org/)), or the [ARRIVE guidelines](http://www.plosbiology.org/article/info:doi/10.1371/journal.pbio.1000412) for reporting work involving animal research. Where applicable, authors should refer to any relevant reporting standards documents in this form.

If you have any questions, please consult our Journal Policies and/or contact us: [editorial@elifesciences.org](mailto:editorial@elifesciences.org).

**Sample-size estimation**

- You should state whether an appropriate sample size was computed when the study was being designed
- You should state the statistical method of sample size computation and any required assumptions
- If no explicit power analysis was used, you should describe how you decided what sample (replicate) size (number) to use

Please outline where this information can be found within the submission (e.g., sections or figure legends), or explain why this information doesn’t apply to your submission:

This information is provided in the Results section and in the first and third paragraphs of ‘Dataset assembly’ in the Methods section. We have also reported the sample size (the number of observations) in figures or their legends.

**Replicates**

- You should report how often each experiment was performed
- You should include a definition of biological versus technical replication
- The data obtained should be provided and sufficient information should be provided to indicate the number of independent biological and/or technical replicates
- If you encountered any outliers, you should describe how these were handled
- Criteria for exclusion/inclusion of data should be clearly stated
- High-throughput sequence data should be uploaded before submission, with a private link for reviewers provided (these are available from both GEO and ArrayExpress)

Please outline where this information can be found within the submission (e.g., sections or figure legends), or explain why this information doesn’t apply to your submission:

This information is provided throughout the Methods section. We reported the detailed information on experimental duration of all studies included in our meta-analysis in Supplementary file 4. We set four criteria, and a total of 284 paired observations in 65 published papers met our criteria and were included in our study (Figure 1; Supplementary file 1). These 284 paired observations were from different combinations of 184 species including 149 tree species, 14 shrub species, and 21 herbaceous species from forests worldwide (Supplementary file 3).

**Statistical reporting**

- Statistical analysis methods should be described and justified
- Raw data should be presented in figures whenever informative to do so (typically when N per group is less than 10)
- For each experiment, you should identify the statistical tests used, exact values of N, definitions of center, methods of multiple test correction, and dispersion and precision measures (e.g., mean, median, SD, SEM, confidence intervals; and, for the major substantive results, a measure of effect size (e.g., Pearson's r, Cohen's d)
- Report exact p-values wherever possible alongside the summary statistics and 95% confidence intervals. These should be reported for all key questions and not only when the p-value is less than 0.05.

Please outline where this information can be found within the submission (e.g., sections or figure legends), or explain why this information doesn’t apply to your submission:

The statistical analysis methods used are described in ‘Meta-analysis’ in the Methods section. Brief summaries of statistical test results including effect sizes, 95% confidence intervals, Pearson's r, and p-values are provided in the Results section, and in figures and their legends, with complete details in the source data tables in Dryad, Dateset (<http://doi.org/10.5061/dryad.nk98sf7qc>).

(For large datasets, or papers with a very large number of statistical tests, you may upload a single table file with tests, Ns, etc., with reference to sections in the manuscript.)

**Group allocation**

- Indicate how samples were allocated into experimental groups (in the case of clinical studies, please specify allocation to treatment method); if randomization was used, please also state if restricted randomization was applied
- Indicate if masking was used during group allocation, data collection and/or data analysis

Please outline where this information can be found within the submission (e.g., sections or figure legends), or explain why this information doesn’t apply to your submission:

This information is provided in the second and third paragraphs of ‘Dataset’ and the first paragraph of ‘Meta-analysis’ in the Method section. The two groups: control (litter decomposing singly) and treatment (litter decomposing in a mixture), and the relevant data were directly obtained from each individual study in this meta-analysis.

**Additional data files (“source data”)**

- We encourage you to upload relevant additional data files, such as numerical data that are represented as a graph in a figure, or as a summary table
- Where provided, these should be in the most useful format, and they can be uploaded as “Source data” files linked to a main figure or table
- Include model definition files including the full list of parameters used
- Include code used for data analysis (e.g., R, MatLab)
- Avoid stating that data files are “available upon request”

Please indicate the figures or tables for which source data files have been provided:

For Figure 1, information on location and climatic variable of studies used in the meta-analysis can be found in Supplementary file 1. For Figure 7, information on divergence in initial chemical traits among component litter species in 284 mixtures can be found in Supplementary file 4. For Figures 1-7, source data files regarding effect size and 95% CI for mass loss or nitrogen and phosphorus release can be found in Dryad, Dateset (<http://doi.org/10.5061/dryad.nk98sf7qc>).
